# The American Medical College Association

**Published:** 1880-11

**Authors:** 


### Editorial.

### THE AMERICAN MEDICAL COLLEGE ASSOCIATION.

In commenting on the position and progress of the above named association, in the JOURNAL AND EXAMINER for October, we alluded to the withdrawal of two of the colleges in New York City, and some reasons assigned for such a course in an editorial article in the *Medical Record* of that city.

The article to which we allude in the *Medical Record*, and from which we quoted freely in our previous number, without directly asserting it, fairly conveyed the inference that the large colleges in the great Eastern cities had either withdrawn from, or refused to co-operate with the College Association for the reason that it "can do *them* no good, and that they do not have faith in the possibility of securing any great reforms in medical education through its influence." The "great reforms" alluded to by the *Record* were, "raising the lecture fees" and "the insisting that a medical college should furnish a certain amount of *clinical* instruction;" and the impression left upon the mind of the reader was, that the colleges in the great Eastern cities had withdrawn or kept aloof from the College Association because a majority of its members being the smaller colleges, "scattered throughout the West and South," it could not possibly adopt these measures of reform without a "fatal loss of membership." In our previous article we showed the fallacy of this pretext, so far as related to the raising of fees, by stating the *fact* that all the important medical colleges in the West (except two State institutions) whose fees were inordinately low, had actually raised them to $75. In regard to the other "great reform," which is a very desirable one, namely, "the insisting that a medical college should furnish a certain amount of *clinical* instruction," we are prepared to show, first, that the colleges in the great Eastern cities have neither insisted that their own students should attend any amount of clinical instruction as a condition for graduation, nor ever asked that the College Association should establish such a rule, and second, that the leading colleges in the West and Southwest are now, and have been, for twenty-five years past, holding a position in regard to clinical instruction in advance of that held by the colleges in the great Eastern cities.

While the annual announcements of the medical colleges in Philadelphia, New York and Boston set forth very prominently the facilities for clinical instruction in the hospitals and dispensaries of those cities, we have failed to find a single one that makes actual *attendance* on such instruction one of the conditions for graduation; and some of them take pains to state positively that such attendance is *not* obligatory. On the other hand, so early as the autumn of 1850, the Rush Medical College of Chicago made attendance on hospital clinical instruction during at least one full college term, one of the requirements for graduation. The Chicago Medical College, now the Medical Department of the Northwestern University, organized in 1859, adopted the same requirement and has enforced it during the whole period of its history. At present, the same requirement is enforced by the Detroit Medical College, the Medical Department of the University of Louisville, and several other schools in the Western cities. Not only have many of the leading medical colleges in the West made attendance on hospital clinical instruction a condition for graduation, but they have more perfectly systematized that important department with a view to having every senior student personally trained in the examination of patients, the handling of instruments for aiding in the diagnosis of diseases, as well as in noting the effects of treatment.

For instance, the Chicago Medical College regularly divides her whole clinical class, composed of the second and third year students, into sub-classes of six students each; and six of these small classes are regularly assigned to as many special departments, among which are the departments of gynæcology, diseases of the eye and ear, diseases of the chest and air passages, diseases of the nervous system, diseases of children, dermatology, etc., in which they are instructed one hour each day for one week, while the remainder of the class are attending the daily general clinics. The next week they fall back into the general clinics and another six of the small classes take their places. This full and carefully matured system of daily clinical instruction has been an essential part of the curriculum of instruction in this college for more than a decade of years. It will be seen by the announcements of the Rush Medical College that similar advantages are afforded for personal clinical instruction in some of the departments, though perhaps less in detail than that just mentioned. We have no hesitation in claiming that there are no hospitals in the great cities of the Eastern part of our country where, during the last quarter of a century, more faithful or systematic clinical instruction has been given at the bedside of the sick, than in those of New Orleans, Louisville, St. Louis, Cincinnati, Chicago and Detroit. And there are no medical colleges in the former cities whose faculties have done as much to make attendance on such clinical instruction a *necessary* part of the student's education, as has been done by the faculties of many of the colleges in the last named cities.

While on the subject of this comparison of the colleges of the East and West, we will go further and remind our Eastern friends of a few historical facts which they will do well to remember. If they will take the trouble to refer to the proper records, they will find that when, in 1866, the American Medical Association had become weary of listening to report after report from the standing committees on medical education, and of adopting resolution after resolution recommending the colleges to adopt a higher standard of requirements for the graduation of students and a better system of college instruction, it was a representative from a *Western* medical school that moved the call for a convention of delegates from medical colleges exclusively to take action on these topics. And when in obedience to the call the college delegates assembled in convention in Cincinnati, May, 1867, there were found to be present representatives from medical colleges in every large city in the West and Southwest, not excepting San Francisco on the Pacific Coast, while not a representative appeared from any college in New York, Brooklyn or Boston. Of the eighteen medical colleges represented in that convention, only four were from colleges in the East, namely one from the University of Pennsylvania and one from the Jefferson College in Philadelphia; one from the Medical College in Albany, N. Y.; and one from the Berkshire College in Pittsfield, Mass.; all the rest being from colleges in the West and South.

In looking further, it will be found that the propositions embodying a full scheme of medical college education, including a fair standard of preliminary education and a full *three years' graded course* of study, of which "clinical medicine and clinical surgery in a hospital," constituted an essential part, were prepared and presented for the consideration of the convention by a delegate from a Western college; that after three days of careful consideration and free discussion, the several propositions were adopted without any essential alterations, without a vote in the negative. Let it be remembered that this was done by a convention of delegates representing *fourteen* medical colleges "scattered throughout the West and South," *two* in *one* of the "great cities of the East," and two in the smaller cities of the East; that it was in 1867; and that the propositions then deliberately adopted embraced fully all the "great reforms" alluded to by the editor of the *Medical Record*, except that of lecture fees. That convention appointed a committee of five, to furnish copies of the propositions adopted to the faculties of all the regular medical colleges in the United States, and ask for their practical adoption. The same committee was authorized to call another convention if, in its correspondence with the medical colleges, such a step should be thought advisable. The committee performed its duties faithfully, and reported the full results of its correspondence with the colleges to a second convention which assembled in the City of Washington in May, 1870.

In this second convention twenty-one medical colleges were represented by accredited delegates. Of these, two were located in Philadelphia and one in Buffalo; the remaining eighteen were "scattered throughout the West and South," but not one in the great cities of New York, Brooklyn and Boston.

And when propositions for re-affirming and adopting the plan agreed upon at the first convention were under consideration and a vote was about to be taken, the question was asked by one of the delegates from a college in St. Louis, whether the action to be taken was to be considered *binding* on the colleges represented or only *recommendatory*, the representatives of the two large schools in Philadelphia, Professors Gross and Stillè, promptly answered that they had *no authority* to bind their respective colleges, while the delegates from many of the leading colleges in the West and South announced that they had come to the convention fully authorized to take such action as would be obligatory upon the colleges they represented. But the entire absence of representatives from the colleges in New York and Boston, with the position taken by those from the colleges in Philadelphia, effectually prevented anything more than a renewal of the *recommendations* made by the convention of 1867. It is thus made apparent by direct historical data, not only, that individual colleges in the West have been much in advance of those in the "great cities of the East" in practically adopting long lecture terms," a full graded three-years course of medical studies, and actual attendance on hospital clinical instruction as a requirement for graduation; but that for fifteen years, at least, a large majority of the medical colleges in the West, from the lakes to the Gulf of Mexico, have been ready to adopt all these improvements with a fair standard of preliminary education added; and have only been waiting for the co-operation of the "larger colleges in the great Eastern cities." Not only is this true as applied to the medical colleges of the West, but it is equally so in regard to the sentiments of the profession at large in the same section, as our friend of the *Medical Record* may find if he chooses to look at the resolutions concerning this subject, adopted by the State Medical Societies of Michigan, Wisconsin, Minnesota, Illinois and Missouri, during the last two or three years. Yet with all the facts to which we have alluded concerning the position of the medical schools and of the profession in the West, on the subject of medical education on record, accessible to any writer who will take the trouble to make the necessary references, Eastern journalists and others continue to represent the scheme of medical college instruction which was established in the Chicago Medical College in 1859; fully discussed and approved by two medical college conventions, one in 1867 and the other in 1870, between which periods it was formally presented for the consideration of the faculties of all the medical colleges in the United States, as the "Harvard Plan," and of Harvard College as *the pioneer* school in adopting it. They excuse the *larger schools* of the *great Eastern cities* for withdrawing or standing aloof from the American Medical College Association because a majority of its members are "smaller colleges scattered throughout the West and South," and *therefore unable* to adopt any great reforms without a "fatal loss of membership." We confess that we are a little out of patience with this persistent misrepresentation of the facts of past history and of present relations—this Pecksniffian assumption that there is nothing of value or importance in the medical institutions of this country outside of that circumscribed strip of territory lying between the eastern part of the Alleghanies and Plymouth Rock.

And having entered our protest in as respectful a manner as possible, we shall in another number of the JOURNAL AND EXAMINER state what we think needs to be done for the best interests of the Association, the profession, and the colleges in all sections of our country.

